# Carbon Dots-AS1411 Aptamer Nanoconjugate for Ultrasensitive Spectrofluorometric Detection of Cancer Cells

**DOI:** 10.1038/s41598-017-11087-2

**Published:** 2017-09-05

**Authors:** Hasan Motaghi, Masoud Ayatollahi Mehrgardi, Philippe Bouvet

**Affiliations:** 1 Department of chemistry, University of Isfahan, Isfahan, 81746-73441 Iran; 2 Université de Lyon, Centre de Recherche en Cancérologie de Lyon, Cancer Cell Plasticity Department, UMR INSERM 1052 CNRS 5286, Centre Léon Bérard, Lyon, France; 3 Université de Lyon, Ecole Normale Supérieure de 3 Lyon, Lyon, France

## Abstract

In the present study, a sensitive and selective signal-on method for aptamer based spectrofluorometric detection of cancer cells is introduced. AS1411, a nucleolin aptamer, is wrapped around water-soluble carbon dots and used as a probe for the detection of several types of cancer cells. Nucleolin, is overexpressed on the surface of cancer cells. Mouse breast 4T1, human breast MCF7, and human cervical HeLa cancer cells were selected as target cells, while human foreskin fibroblast cells HFFF-PI6 served as control cells. For the sensitive and selective spectrofluorimetric detection of target cancer cells in the presence of control cells, the cells were incubated in carbon dots-aptamer solutions, the cell suspensions were subsequently centrifuged and the fluorescence intensities were measured as an analytical signal. The specific targeting of cancer cells by AS1411 aptamers causes the release of carbon dots and enhances the fluorescence intensity. A calibration curve with a dynamic range between 10–4500 4T1 cells and detectability of roughly 7 cells was obtained. In addition, no significant change in the signal was detected by modifying the amount of human foreskin fibroblast control cells. Our results demonstrate similar responses to human MCF7 breast and cervical HeLa cancer cells.

## Introduction

Cancer is a major cause of mortality worldwide and its early diagnosis significantly increases patient survival rates^1^. Most biochemical analysis techniques employed to detect cancer cells are based on the use of specific ligands for protein recognition. For instance, aptamers and proteins, including antibodies and enzymes, have been used for the detection of cancer cells, due to their specificity and high binding affinity^2^. Furthermore, several labeling techniques, such as fluorescent^3^, chemiluminescent^4^, radioactive^5^ and electrochemical^6–8^ labeling have been developed for cancer cell detection at the molecular level. However, applications for such methods remain limited owing to their elevated cost and complexity.

Nucleic acid aptamers are single-stranded DNA or RNA that specifically recognize their target and are very often identified from random sequence libraries by systematic evolution of ligands by exponential enrichment (SELEX). Aptamers are acknowledged as promising alternatives to antibodies in protein recognition and sensing, owing to their simple synthesis, easy storage, excellent controllability and broad applicability^9^. Furthermore, they form well-ordered structures, with high affinity and specificity. They can bind various targets, such as inorganic ions, small molecules, proteins and even whole cells^10–12^.

AS1411 is a 26-mer oligonucleotide that targets nucleolin^13, 14^. Nucleolin is a multifunctional protein located primarily in the nucleolus, but is also found in the cytoplasm and on the membrane of cells^14, 15^. AS1411 binds to nucleolin with high affinity, though this mechanism of interaction is poorly understood. This protein is overexpressed in many types of tumor cells compared to normal cells, and cancer cells consequently display a higher amount of nucleolin on their surface. It was also reported that AS1411 initially binds to nucleolin on the surface of tumor cells prior to being taken up by the cells^16^.

Aptamer-based spectrofluorometric assays offer one of the most sensitive protocols for the detection of cancer cells^17–21^. The efficiency of spectrofluorometric protocols can be further improved by the use of nanostructures, as evidenced by the successful application of aptamer-conjugated fluorescence silica nanoparticles^18^, CdSe/ZnS core/shell quantum dots^22^ and carbon nanodots^19, 21^ for the sensitive monitoring of cancer cells.

Quantum dots (QDs) and organic dyes are used as fluorophores in fluorescent methods^23^. Recently, carbon nanoparticles under 10 nm in size, also known as carbon dots (CDs), were used as highly efficient fluorophores^24^. They were shown to offer several advantages compared to traditional fluorescent labels such as suitable photostability, favorable biocompatibility, low toxicity, high water solubility, broad excitation spectrum, appropriate quantum yield (QY) and resistance to photobleaching, which makes them interesting candidates for biological experiments^25, 26^. Furthermore, CDs can be easily functionalized due to the presence of various functional groups on their surface, depending on their precursors^27^. Different methods of CDs synthesis, such as thermal pyrolysis^28^ and combustion/thermal microwave heating^29, 30^, laser ablation^31^ and electrochemical oxidation^32^ have been reported in the literature. Among these methods, hydrothermal synthesis is favored due to its simplicity and lower cost.

 In the present manuscript, mouse breast tumor cells (4T1), human breast tumor cells (MCF7), and human cervical cancer cells (HeLa), all of which overexpress nucleolin on their surface, were incubated in the presence of control human foreskin fibroblast cells (HFFF-PI6) and CDs-AS1411 aptamer probes to investigated the sensitivity and selectivity of our signal-on spectrofluorometric assay for the targeted detection of cancer cells.

## Results and Discussion

**Figure 1:**
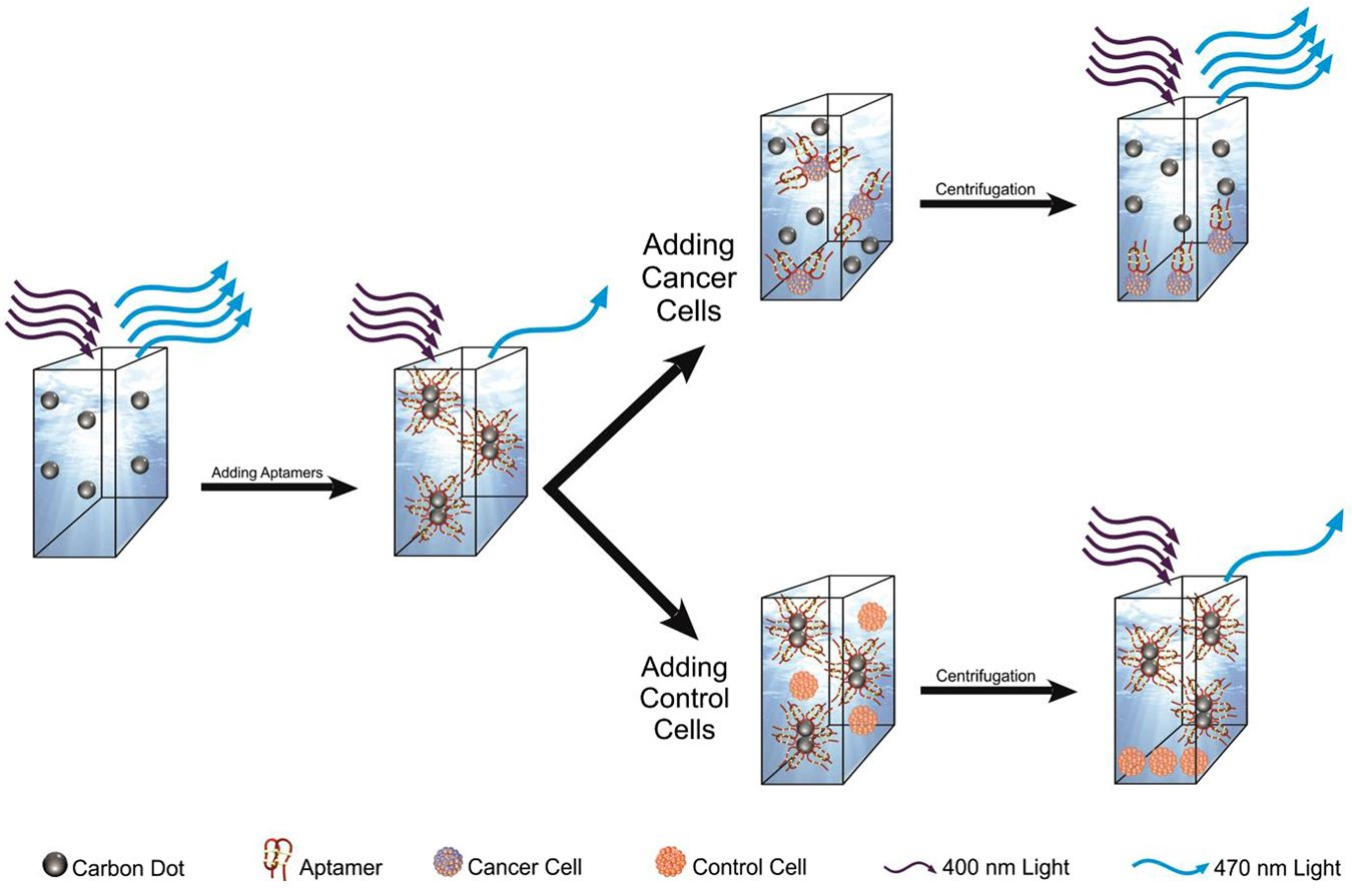
Schematic representation of the principle of cancer cell detection using the carbon dots-AS1411 aptamer nanoconjugates.

The principle of our spectrofluorometric method is described in Fig. 1. Briefly, CDs emit a blue fluorescence (470 nm) under UV (400 nm) light, the intensity of which decreases once AS1411 aptamers wrap around them. In presence of cancer cells overexpressing nucleolin, the preferential interaction between the aptamer and nucleolin causes its release from CDs. The subsequent centrifugation of the suspension of cancer cells, CDs and aptamers, leads to the precipitation of cancer cell/nucleolin-aptamer conjugates and to the re-emission of CD fluorescence in the supernatant which can then be measured. Inversely, upon addition of control cells, no interaction between cells and aptamers is expected to occurs, and then the fluorescence intensity of the supernatant should remains unaltered.

### Synthesis, characterization, and optical behavior of carbon dots

**Figure 2:**
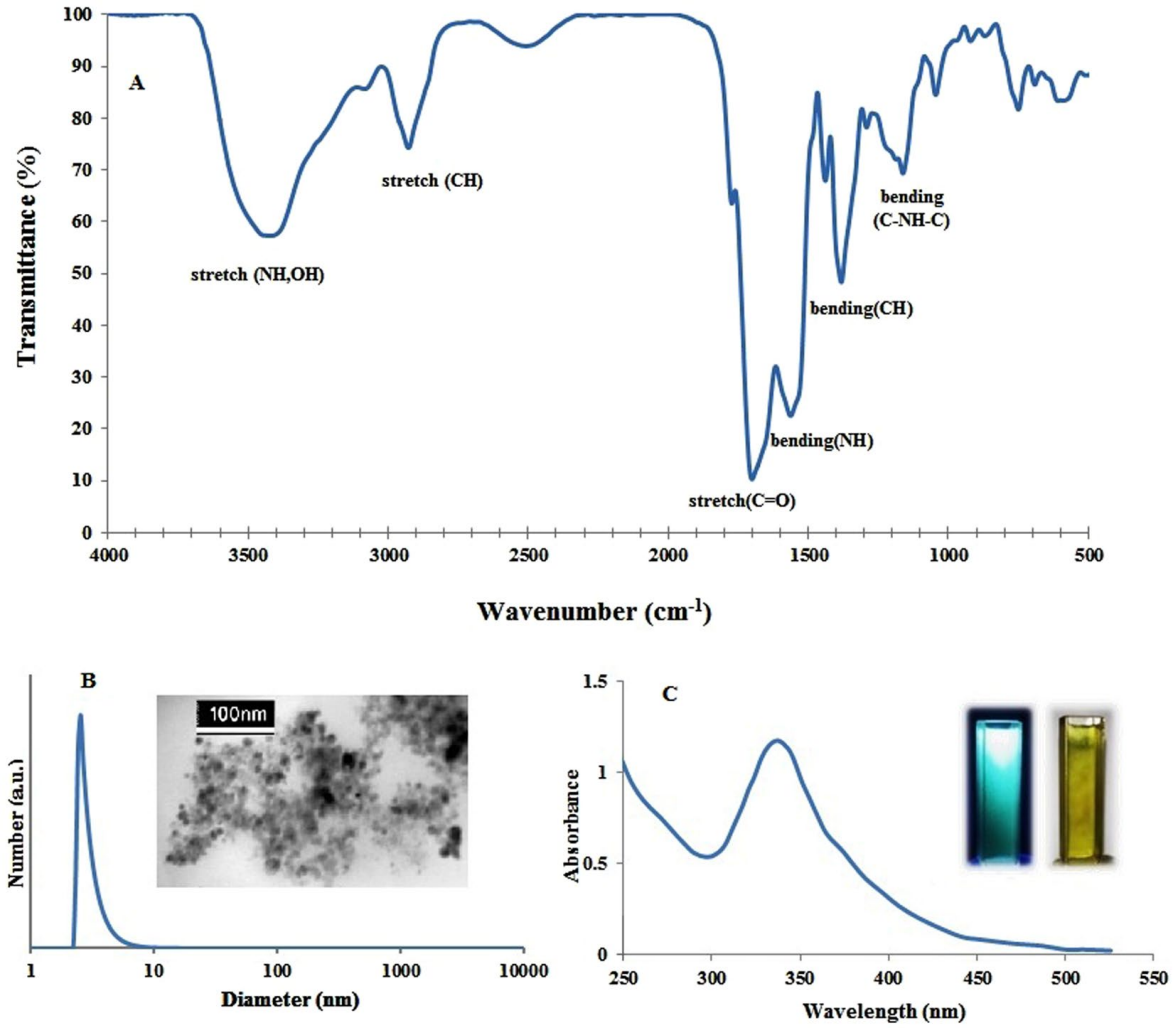
FTIR spectrum (**A**), dynamic light-scattering spectrum (**B**), transmission electron microscopy image (B-inset) and ultraviolet-visible (UV/Vis) absorption spectrum (in aqueous solution) of CDs, the photograph of the CDs under sunlight and hand-held UV light (**C**-inset from left to right).

As mentioned in the experimental section, CDs were synthesized by hydrothermal method as previously described by Zhu *et al*.^33^. The Fourier transform infrared spectroscopy (FTIR) spectrum obtained confirmed the presence of several functional groups, such as -NH_2_ and - COOH, on the surface of CDs (Fig. 2A). These functional groups impart excellent properties to CDs rendering them more dispersible in water, and provide good platforms for their conjugation with biomolecules. Transmission electron microscopy (TEM) images and dynamic light scattering (DLS) results demonstrated that CDs are generally uniformly dispersed with particle diameters of ~2.5–10 nm (Fig. 2B). Of note, CD suspensions were centrifuged at 10,000 rpm before recording these images.

**Figure 3:**
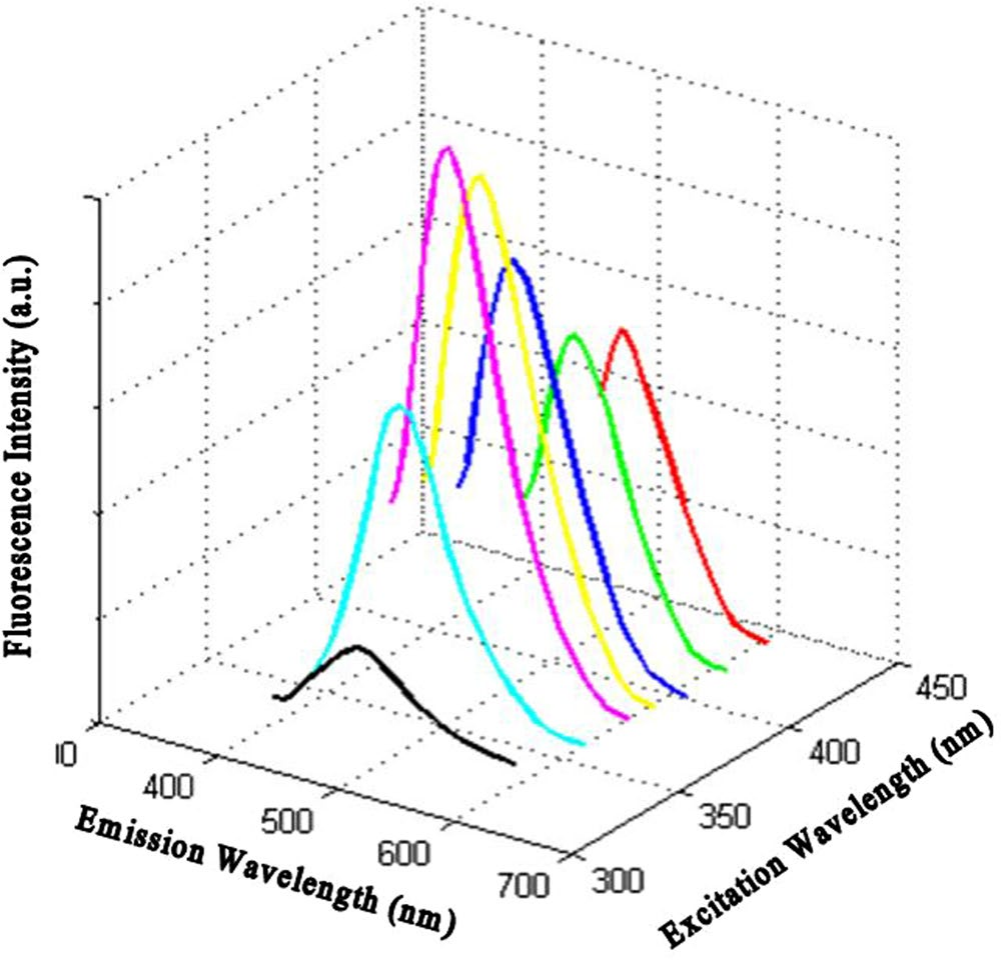
Photoluminescence spectra of the CDs at different excitation wavelengths.

As Fig. 2C illustrates, the UV-Vis spectrum of CDs shows a broad absorption band with a maximum absorbance peak at 340  nm which is consistent with values obtained for CDs synthesized according to the same protocol^33^. The photographs of CDs under hand-held UV light and sunlight (Fig. 2C, inset), illustrate the intense blue fluorescence emitted under UV light. Furthermore, the fluorescence spectra at different excitation wavelengths (Fig. 3), clearly demonstrate an excitation-dependent photoluminescence behavior which is very common for fluorescent CDs^34^. The synthesized CDs also displayed a good QY of 0.54, indicating that CDs are suitable candidates as cheap fluorophores. We have herein chosen a maximum emission wavelength of 470 nm with an excitation at 400 nm for florescence intensity measurements.

### Optimization of experimental conditions

Since ethylene diamine was one of the precursors for the synthesis of CDs and considering the FTIR spectrum shown in Fig. 2A, amine groups are present on CD structures and develop positive charges on their surfaces. Therefore, the electrostatic interaction between positively charged CDs and negatively charged DNA aptamers led to the wrapping of aptamers around CDs. Furthermore, the aptamer acted as a bridge between CDs through the formation of amide bonds between carboxyl or amine groups on CDs and amine or phosphate groups of the aptamers, respectively, which are physisorbed onto the surface of CDs^35^. The reduction in fluorescence following the wrapping of the aptamer around CDs demonstrates that aptamer molecules can partially quench the fluorescence of CDs^36^. This can be attributed to aptamer-CDs interactions that bring nanodots closer to each other^37, 38^ which can lead to self-quenching ascribed by excessive resonance energy transfer (RET) or direct π-π interactions^39^. The kinetics for this phenomenon is relatively slow as it takes time for effective interactions to occur between them. By increasing incubation time up to 1 hour, fluorescence intensities decreased, though no additional quench was observed after 1 hour (Figure S1). The comparison between UV-Vis spectra of CDs, aptamer, and CD-aptamer nanoconjugate suspensions confirmed the interaction between CDs and aptamers (Figure S2).

The maximum difference between fluorescence intensities in the presence and absence of cancer cells is vital to obtain the best analytical performances. By adding different concentrations of aptamers to CDs (up to 4 μM of aptamers) in the absence or presence of the 4T1 cells, we identified an optimum change in fluorescence intensity at an aptamer concentration of 1 μM, followed by a gradual decrease (Figure S3). This can be attributed to the intramolecular interaction of aptamer strands in the presence of excessive amounts of aptamers, which leads to a reduction in the functionality of aptamers.

**Figure 4:**
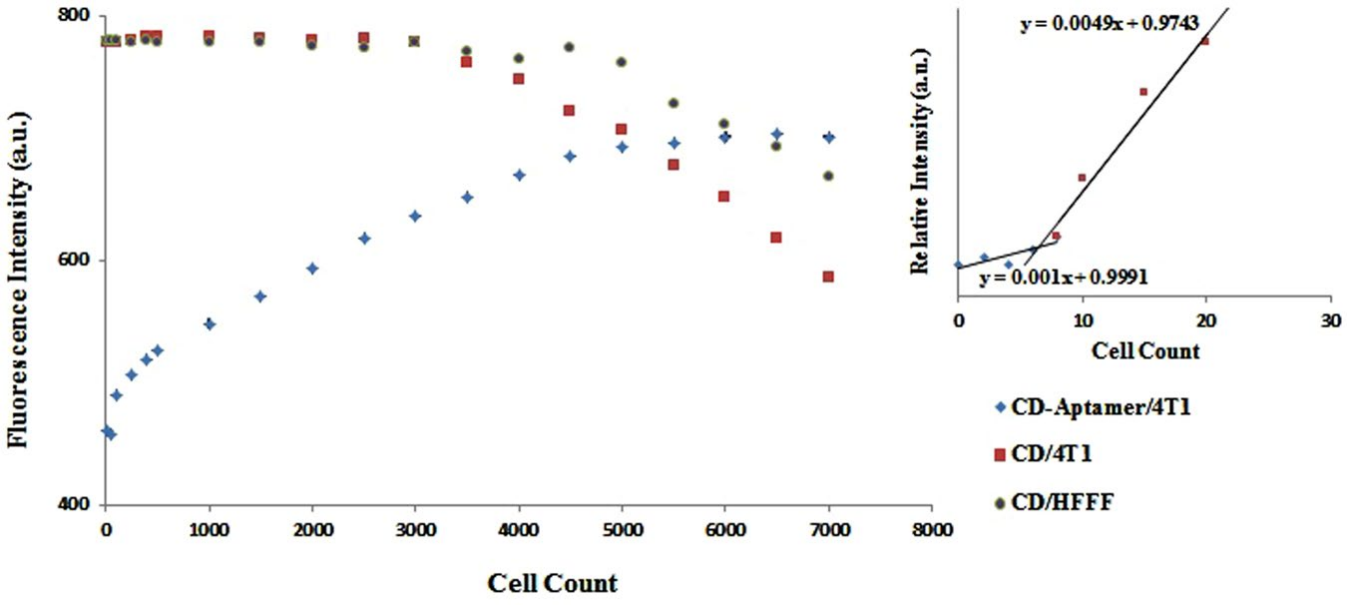
The fluorescence intensity of CDs and CDs-aptamer nanoconjugates vs. cell count of 4T1 (cancer) and HFFF (control) cells. Inset: The calibration curve for 4T1 cells. The detection threshold was determined by taking the intersection of the extrapolated linear sections of the calibration curve.

**Figure 5:**
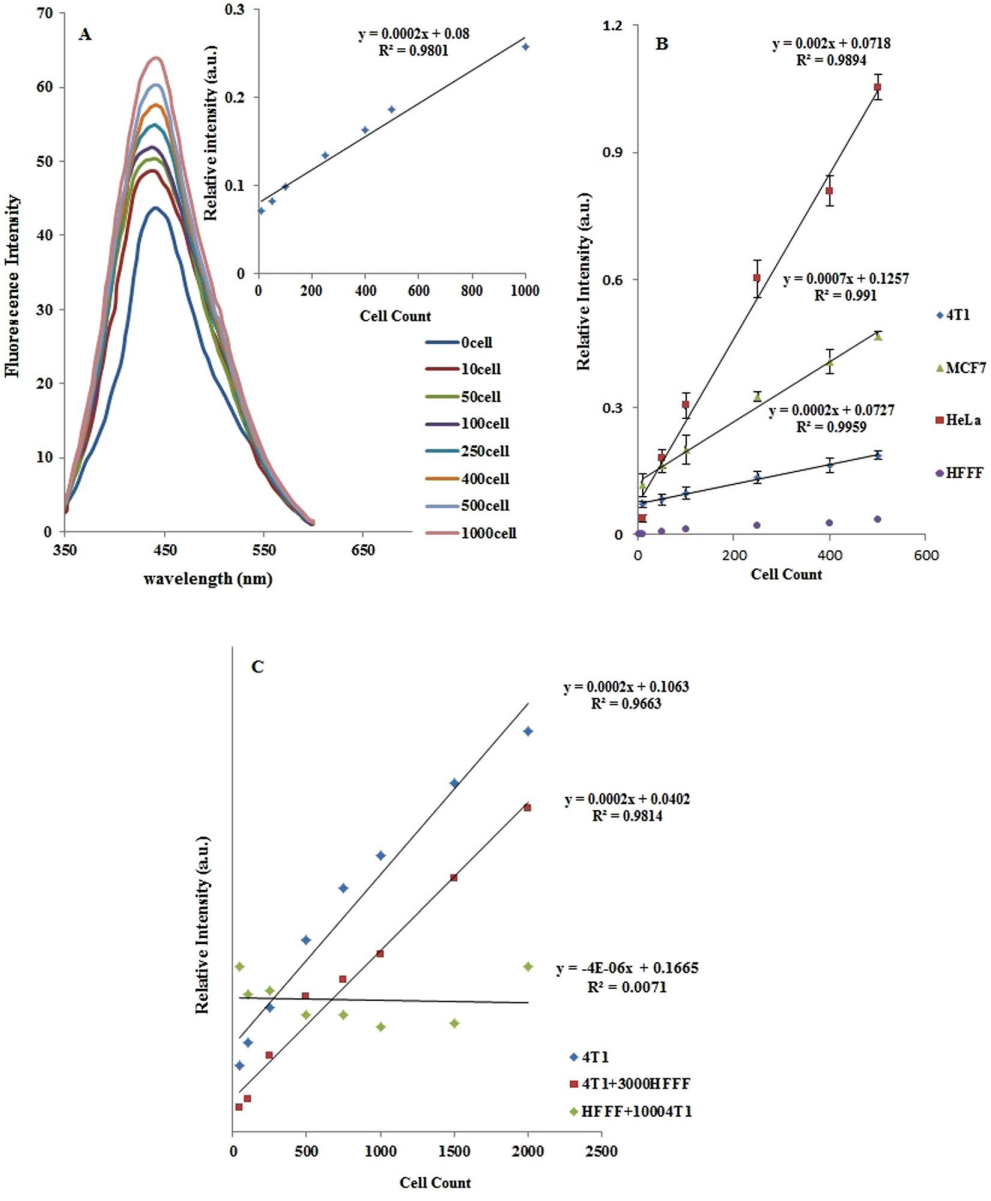
Fluorescence spectra of samples with 0–1,000 4T1 cells (**A**), calibration curves of 4T1 cells (**A**-inset), calibration curves of MCF7, HeLa and 4T1 cancer cells, as well as HEFF cells used as control cells (**B**), calibration curve of a combination of 0–2,500 4T1 cells and 3,000 HFFF cells or of 1,000 4T1 cells and 0–2,500 HFFF cells (**C**).

We next determined the effect of incubating mouse breast tumor cells (4T1) with CD-aptamer on the fluorescence of CDs. As these cancer cells express nucleolin on their surface, there are expecting to interact with the aptamer attached to the CDs (Fig. 1). Indeed, incubation of 4T1cells with CDs-aptamer nanoconjugates abolished the quenching observed for CDs conjugated with aptamers (Fig. 4). This reaction took place within 5 minutes (including incubation time and centrifugation step) and was sufficient to obtain the maximum difference in relative intensity. These intensities remain constant for up to 25 minutes, after which time the intensities diminished. Higher fluorescence intensities within the first 25 minutes of incubation can be attributed to the release of CDs from aptamer molecules due to the high affinity and specificity of the AS1411 aptamer for nucleolin. This phenomenon also indicates that aptamer molecules are wrapped around CDs physically and they can easily be unwrapped in the presence of the cancer cells. In contrast after longer incubation times, the fluorescence intensities significantly decreased probably due to nonspecific interactions between CDs and cells or to internalization of CDs by cells (Figure S4). Figure S5 shows the fluorescence microscopic image of 4T1 cells after 180 minutes and confirms the uptake of CDs after a long incubation time. But after a 15 minutes incubation time, no fluorescence could be detected within the cells indicating that during this short incubation time no significant internalization of CDs is taking place. Therefore, a 15 minutes incubation time was chosen for further experiments. Inversely, our findings demonstrated no significant change in the fluorescence intensity of CDs-aptamer suspensions over time in the presence of control cells under similar experimental conditions (Fig. 5).

### Carbon dots as excellent fluorophores for the sensitive detection of cancer cells

Next, based on the optimized experimental conditions mentioned in the previous section, the performance of the assay on different cell lines was investigated. For this purpose, 20 μL CDs-aptamer suspensions, at an optimum CD/aptamer ratio, were incubated with various concentrations of 4T1 cells for 15 minutes. The suspensions were centrifuged and the fluorescence intensities recorded before and after incubations. A calibration curve with a dynamic range of 10–4500 4T1 cells in 60 µL was plotted to measure the relative fluorescence intensities as a function of cell numbers. The detection threshold was obtained by plotting the intersection of the extrapolated linear sections of the calibration curve. A minimum detectability of roughly 7 cells in 60 µL sample (105 cells/mL) was obtained (Fig. 4, inset). The calibration curve displayed a plateau at higher concentrations. The fluorescence intensities of CDs alone, before and after incubation with cancer (4T1) and control human foreskin fibroblast cells (HFFF), revealed that there was no significant change in relative intensity up to 4,000 cells, but at higher concentrations, this value decreased for both target and control cells, indicating either a nonspecific uptake of CDs by cells in the absence of aptamers by both cell lines or the aggregation of CDs (Fig. 4).

To investigate the performances of the protocol for other nucleolin positive cells, the CDs-aptamer suspensions were incubated with different concentrations of various cancer cells, including the 4T1 cells, the human breast cancer cell line (MCF7) and the human cervical cancer cell line (HeLa). The HFFF cells served as control cells. As shown in Fig. 5, the calibration sensitivity for HeLa cells was higher than that for MCF7 cells and the protocol for MCF7 was more sensitive than with 4T1 cells. These differences in sensitivity of CDs-AS1411 aptamer quenching according to the cell type could be dependent on the level of nucleolin expression on the surface of the cells. Although it has not been experimentally demonstrated that HeLa cells have more surface nucleolin than MCF 7 and 4T1, our data suggest that this is the case. Furthermore, we cannot exclude that all of these cells express the same amount of surface nucleolin, but that the accessibility of surface nucleolin for aptamer-nucleolin interactions is different depending on the type of cells. The fluorescence intensity changes for two series of combinations of 4T1 and HFFF cells was also monitored. As shown in Fig. 5C, the analytical signals for 1,000 4T1 cells/mL were not significantly altered in the presence of various amounts of HFFF control cells. In addition, the calibration curves obtained for different amounts of 4T1 in the presence of 3,000 HFFF cells/mL and in the absence of HFFF are identical. As shown by analysis of variance results (ANOVA) in Table S1, the significant differences between the recorded fluorescence intensities for the cancer and normal cells have been observed. It also demonstrates significant differences between the fluorescence of suspensions in the presence of different number of cells.

## Conclusion

Taken together, the assay shows a good sensitivity and a low detection threshold, and is also less time-consuming and easier to implement for the detection of various cancer cells in comparison to previous studies using other nanoparticle devices^35–40^. For example, a detection limit of 250 cells/mL was obtained when using aptamer-conjugated fluorescent Ru(bpy)_3_^2+^ dye-doped nanoparticles^40^ and 50 cells/mL was reported for CCRF-CEM^41^ and Ramos^42^ cells by applying CdSe QDs. Methods with similar or lower detection thresholds have also been reported, including a detection range of over 10 to 10^5^ HeLa cells/mL^43^ using an impedimetric approach and the fluorescence detection of about 6 CCRF-CEM cells^44^. Previously, our research group described an electrochemical aptamer based biosensor for the detection of human liver hepatocellular carcinoma (HepG2) with detection threshold of 2 cells/mL^45^. However, the present protocol offers a very simple and a relatively cheap approach with an acceptable sensitivity and detection threshold of ~100 cells/mL.

## Methods

### Materials and apparatus

Sodium hydroxide, sodium chloride, potassium chloride, sodium dihydrogen phosphate, potassium dihydrogen phosphate, disodium hydrogen phosphate, were purchased from Merck (Darmstadt, Germany). Citric acid, ethylene diamine, fetal bovine serum (FBS), penicillin, streptomycin and Roswell Park Memorial Institute (RPMI) 1640 medium were obtained from Sigma (Sigma-Aldrich, USA). All reagents were of analytical grade and used as received, without any further purification. The AS1411 aptamer described in the literature^46^ with the GGT GGT GGT GGT TGT GGT GGT GGT GGT TT sequence was purchased from Eurofins MWG/Operon (Germany). The stock solutions of the aptamer (100 µM) were prepared using PBS 1x (pH 7.4) and were stored at −20 °C. Distilled deionized water was used in all solution preparations.

Various cell lines including, mouse breast cancer (4T1), human breast adenocarcinoma (MCF7), human cervical cancer (HeLa) and human foreskin fibroblast (HFFF-P16) were obtained from the National Cell Bank of Iran (Pasteur Institute of Iran). The cells were cultured in RPMI-1640 medium supplemented with 10% fetal bovine serum (FBS), penicillin (100  U/mL), and streptomycin (100 µg/mL) and incubated at 37 °C in a humidified incubator with 5% CO_2_. The cells were centrifuged and subsequently re-suspend in the oxygenated sterile PBS, for spectrofluorometric experiments.

Optical absorption spectra were recorded using an UV-Vis spectrophotometer (Shimadzu UV-160). The fluorescence intensities of the solutions were monitored using the Shimadzu RF-5301PC Spectrofluorometer. Transmission electron microscopy (TEM) images were captured on a Philips CM30 TEM. Dynamic light scattering (DLS) measurements were performed by Vascov/Cordouan Technologies (France). Fourier transform infrared spectroscopy (FTIR) spectra were recorded using a FTIR spectrophotometer model JASCO (Japan). The fluorescence microscopic images were captured using Olympus fluorescent microscope (IX71, Japan).

###  Synthesis and characterization of carbon dots

CDs were prepared following a hydrothermal method as previously described by Zhu *et al*.^33^. Briefly, 4 g of citric acid and 1 mL ethylene diamine were dissolved in 40 mL water. The solution was then transferred to a Teflon-lined autoclave and heated to 250 °C for 5 hours. The autoclave was cooled down to room temperature. The resulting transparent brown-black solution was centrifuged at 7,000 rpm for 15 minutes to remove the large black aggregates. The supernatant was kept in dark and used as CD suspension for further experiments and chracterizations. DLS and TEM techniques were used for determining the size of CDs. The fluorescence quantum yield (QY) of CDs was determined using the reference point method by comparing the excitation and emission of CDs with fluorescein as a standard fluorescent compound^47^.

### Preparation of carbon dots-aptamer nanoconjugates

To prepare CDs-aptamer nanoconjugates and for the optimization of the experimental conditions, different volumes of 10  µM aptamer and 10 μL CDs (1.4 mg/mL) in 1x PBS were mixed together and total volumes were fixed at 60 µL. The suspensions were maintained in a dark place for 90 minutes and the fluorescence intensities of these suspensions were then recorded.

### Spectroflurometric monitoring of cells

60 μL aliquots of the cell suspensions including, either various amounts of cancer cells (4T1, MCF7 or HeLa) or control cells (HFFF) or the mixture of cancer and control cells (4T1 + 3,000 cells/mL HFFF or HFFF + 1,000 cells/mL 4T1) were added to the prepared CDs-aptamer suspensions with different CDs/aptamer ratios. After 15 minutes, the suspensions were centrifuged at 4,000 rpm for 5 minutes and the fluorescence intensities of the supernatants were followed and compared with the fluorescence intensities of CDs-aptamer conjugates in the absence of cancer cells.

### Fluorescence Microscopic imaging

The flurescence microscopic images of 4T1 cells were recorded after 15 and 180 minutes incubation of the cancer cells with CDs-aptamer nanoconjugate.

## Electronic supplementary material

Supplementary Information
