# Supplementary material for: Carbon Dots-AS1411 Aptamer Nanoconjugate for Ultrasensitive Spectrofluorometric Detection of Cancer Cells
Source: Sci Rep. 2017 Sep 5;7:10513. doi: 10.1038/s41598-017-11087-2 (PMC5585388; doi:10.1038/s41598-017-11087-2)
Supplement: Supplementary file 1 — Supplementary Information [file 41598_2017_11087_MOESM1_ESM.doc]

## Supplementary Information

## Carbon Dots-AS1411 Aptamer Nanoconjugate for Ultrasensitive Spectrofluorometric Detection of‎ Cancer Cells

Hasan Motaghi^a^, Masoud Ayatollahi Mehrgardi*^,a^, Philippe Bouvet^b^

1. *Department of chemistry, University of Isfahan, Isfahan, 81746-73441, Iran*
2. *Université de Lyon, Ecole Normale Superieure de Lyon, Centre de Recherche en Cancérologie de ‎Lyon, Cancer Cell Plasticity Department, UMR INSERM 1052 CNRS 5286, Centre Léon Bérard, ‎Lyon, France.‎*

∗ Corresponding author. Tel.: +98 311 7932710; fax: +98 311 6689732. E-mail addresses: [m.mehrgardi@sci.ui.ac.ir](mailto:m.mehrgardi@sci.ui.ac.ir), m.mehrgardi@gmail.com (M.A. Mehrgardi).


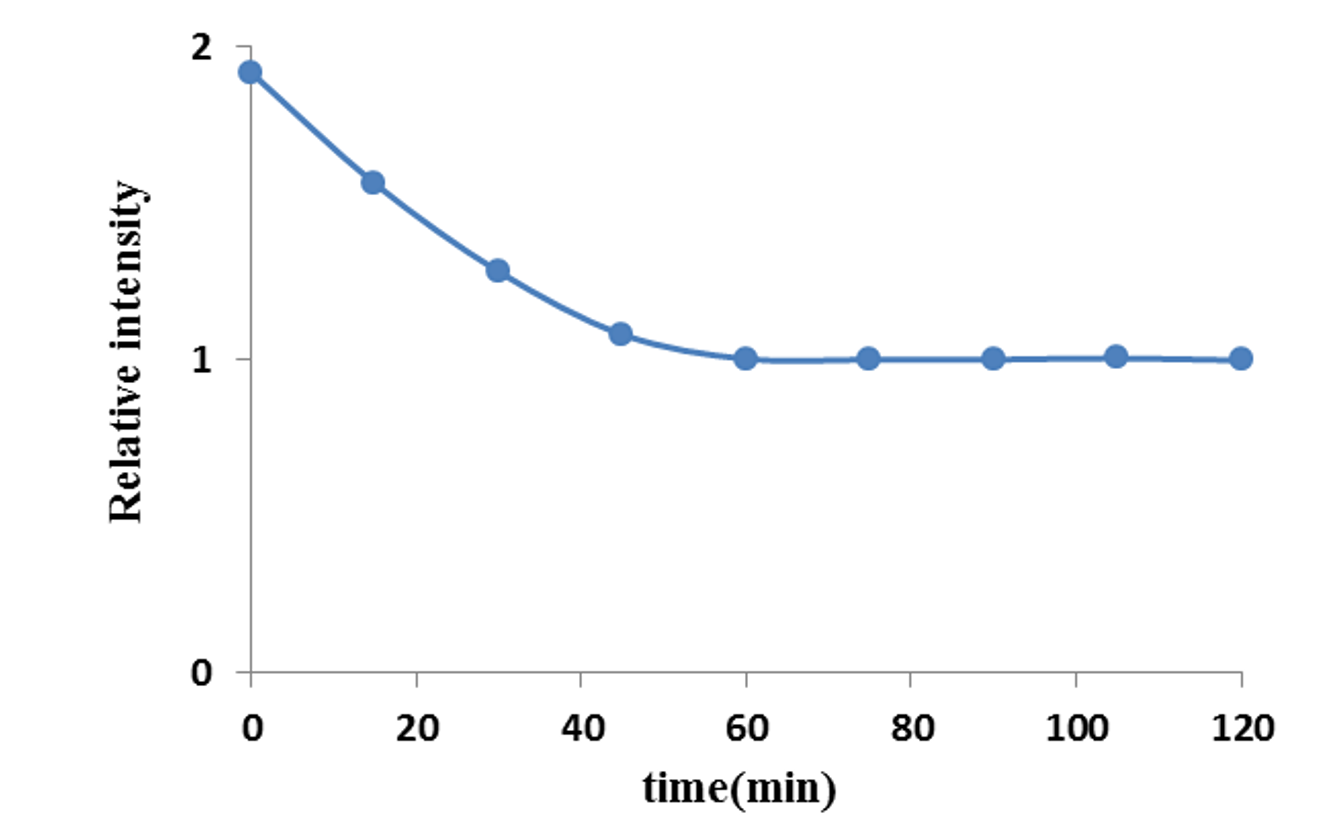


**Figure S1.** In order to find the optimum binding time of CDs and aptamer, 10µL of CD suspension and 1µM of aptamer, was incubated for different times between 5 to 120 min ‎and fluorescence intensity was then recorded. The optimum time for effective interaction and connection is about 1hr. Longer mixing time does not have a ‎significant effect on the fluorescence intensity of solution. The changes in fluorescence intensity ‎during the first hour is a sign of interaction between the CDs and apamer.


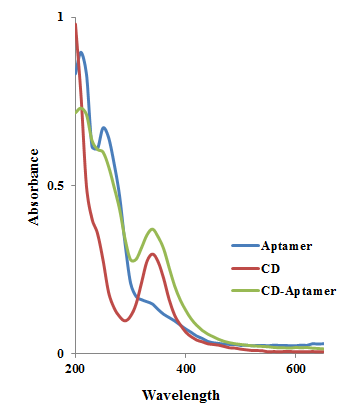


**Figure S2.** Ultraviolet-visible (UV/Vis) absorption spectrum (in aqueous solution) of CDs, Aptamer and CD-Aptamer nano conjugate.


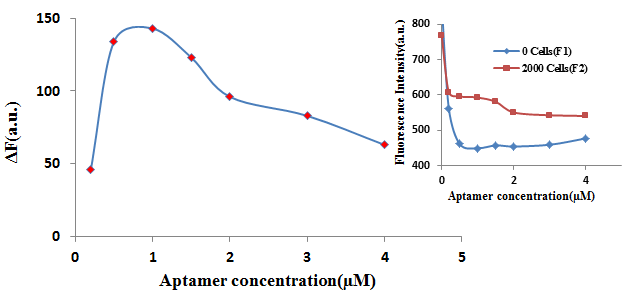


**Figure S3.** Change of flurescence intensity‎ in absence and presence of 4T1 cells (ΔF)‎ versus aptamer concentration reaches a pick at ‏1‏ µM ‎.


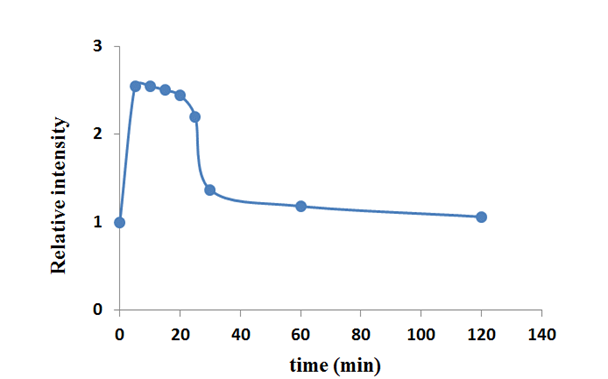


**Figure S4**. Solutions with 2000 4T1 cells were mixed with CD - aptamer nanocanjugate and incubated for 5 to 120 min to find optimum incubation time. After incubation ,the suspensions were centrifuged to remove cells .


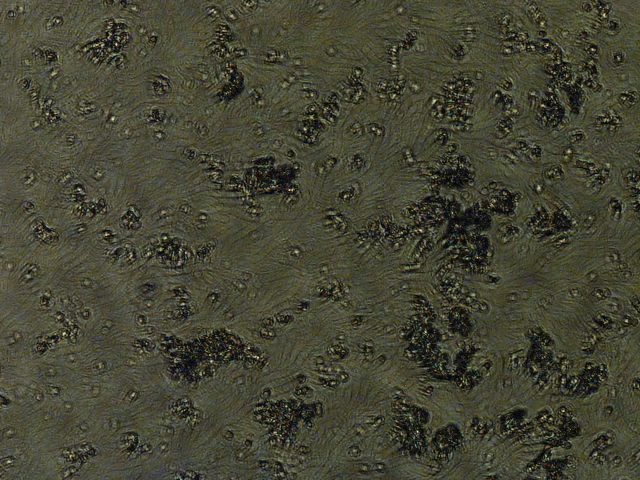

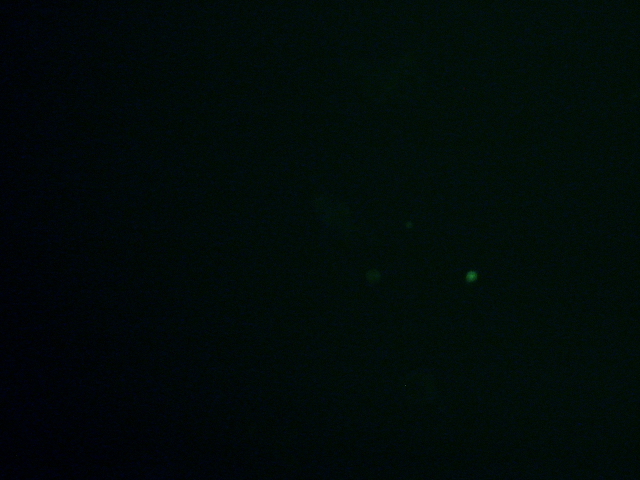

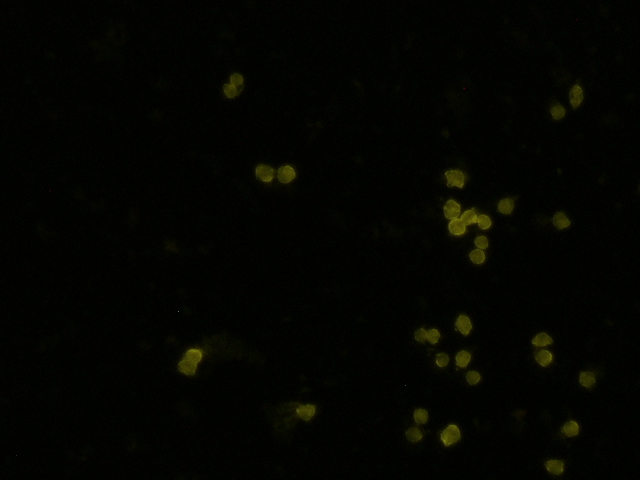


**Figure S5.** a) The optical microscopic image and the fluorescent microscopic images of 4T1 cells after b) 15 min and c) 180 minutes incubation with CD-aptamer nanocanjugate.

**Table S1.** ANOVA table investigating the effect of type of cells (cancer and control) and number of cells.

|  | Anova: Two-Factor With Replication | | | |  |  |  |  |  |
| --- | --- | --- | --- | --- | --- | --- | --- | --- | --- |
|  |  |  |  |  |  |  |  |  |  |
|  | SUMMARY | 10 | 50 | 100 | 250 | 400 | 500 | Total |  |
|  | *4T1* |  |  |  |  |  |  |  |  |
|  | Count | 3 | 3 | 3 | 3 | 3 | 3 | 18 |  |
|  | Sum | 0.215247 | 0.248879 | 0.295964 | 0.403587 | 0.491031 | 0.558296 | 2.213004 |  |
|  | Average | 0.071749 | 0.08296 | 0.098655 | 0.134529 | 0.163677 | 0.186099 | 0.122945 |  |
|  | Variance | 4.66E-05 | 0.000163 | 0.000216 | 0.000186 | 0.000272 | 0.0001 | 0.001985 |  |
|  |  |  |  |  |  |  |  |  |  |
|  | *HFFF* |  |  |  |  |  |  |  |  |
|  | Count | 3 | 3 | 3 | 3 | 3 | 3 | 18 |  |
|  | Sum | 0.087444 | 0.080717 | 0.127803 | 0.154709 | 0.168161 | 0.181614 | 0.800448 |  |
|  | Average | 0.029148 | 0.026906 | 0.042601 | 0.05157 | 0.056054 | 0.060538 | 0.044469 |  |
|  | Variance | 0.000146 | 0.000202 | 0.000154 | 0.000113 | 0.000139 | 4.01E-05 | 0.000268 |  |
|  |  |  |  |  |  |  |  |  |  |
|  | *Total* |  |  |  |  |  |  |  |  |
|  | Count | 6 | 6 | 6 | 6 | 6 | 6 |  |  |
|  | Sum | 0.302691 | 0.329596 | 0.423767 | 0.558296 | 0.659193 | 0.73991 |  |  |
|  | Average | 0.050448 | 0.054933 | 0.070628 | 0.093049 | 0.109865 | 0.123318 |  |  |
|  | Variance | 0.000622 | 0.001088 | 0.001091 | 0.002185 | 0.003639 | 0.004786 |  |  |
|  |  |  |  |  |  |  |  |  |  |
|  |  |  |  |  |  |  |  |  |  |
|  | ANOVA |  |  |  |  |  |  |  |  |
|  | *Source of Variation* | *SS* | *df* | *MS* | *F* | *P-value* | *F crit* |  |  |
|  | Sample | 0.055425 | 1 | 0.055425 | 374.0017 | 3.83E-16 | 4.259677 |  |  |
|  | Columns | 0.026675 | 5 | 0.005335 | 35.99915 | 2.15E-10 | 2.620654 |  |  |
|  | Interaction | 0.008069 | 5 | 0.001614 | 10.8893 | 1.48E-05 | 2.620654 |  |  |
|  | Within | 0.003557 | 24 | 0.000148 |  |  |  |  |  |
|  |  |  |  |  |  |  |  |  |  |
|  | Total | 0.093725 | 35 |  |  |  |  |  |  |
|  |  |  |  |  |  |  |  |  |  |

**Table S1.** ANOVA table investigating the effect of type of cells (cancer and control) and number of cells.
